# Cognitive impairment following traumatic brain injury in Uganda: Prevalence and associated factors

**DOI:** 10.1371/journal.pgph.0001459

**Published:** 2023-02-06

**Authors:** Timothy Mwanje Kintu, Vanessa Katengeke, Ronald Kamoga, Tricia Nguyen, Josephine Nambi Najjuma, David Kitya, Edith K. Wakida, Celestino Obua, Godfrey Zari Rukundo

**Affiliations:** 1 Faculty of Medicine, Mbarara University of Science and Technology, Mbarara, Uganda; 2 Office of Research Administration, Mbarara University of Science and Technology, Mbarara, Uganda; 3 California University of Science and Medicine, Colton, California, United States of America; 4 Department of Neurosurgery, Mbarara University of Science and Technology, Mbarara, Uganda; 5 Department of Medical Education, California University of Science and Medicine, Colton, California, United States of America; 6 Department of Psychiatry, Mbarara University of Science and Technology, Mbarara, Uganda; Bangladesh University of Health Sciences, BANGLADESH

## Abstract

**Background:**

As the burden of dementia continues to rise in sub-Saharan Africa, it is crucial to develop an evidence base for potentially modifiable risk factors such as Traumatic Brain Injury (TBI). Cognitive impairment may result from TBI and since it is an established prodromal form of dementia, we investigated the burden of cognitive impairment and associated factors in persons with a history of TBI in southwestern Uganda.

**Methods:**

This was a community-based quantitative study with a cross-sectional design among 189 persons with a history of TBI in southwestern Uganda. Data were collected by the research team in March and June 2022 and entered into Kobo Toolbox before being transferred to RStudio version 4.1.0 for cleaning and analysis. Data were analyzed at a 5% level of significance.

**Results:**

Most study participants had some form of cognitive impairment (56.1%), with 43.1% of the participants having mild cognitive impairment (MCI). Cognitive impairment was associated with older age (p-value<0.001); loss of consciousness following the TBI (p-value = 0.019) and a history of tobacco use (p-value = 0.003). As a measure of severity of the TBI, loss of consciousness (aOR = 4.09; CI = 1.57–11.76; *p*<0.01) and older age (aOR = 1.04; CI = 1.01–1.07; *p*<0.01) were identified as risk factors for cognitive impairment.

**Conclusion:**

There is a high burden of cognitive impairment among individuals with a history of TBI in southwestern Uganda, and most associated risk factors are potentially modifiable. Long-term follow-up of TBI patients would enable early identification of some risks. Patients with TBI could benefit from behavioural modifications such as restriction of alcohol intake and tobacco use to slow down the progression into dementia.

## Introduction

Traumatic brain injury (TBI) is a common occurrence all over the world, with the incidence of TBI in sub-Saharan Africa (SSA) known to be higher than the global incidence [1]. TBI is a leading cause of morbidity and mortality worldwide [2], and is especially common among young adults [3]. It has been identified as one of the most established risk factors for neurologic and psychiatric illnesses including dementia [4]. One of the consequences of TBI is Mild cognitive impairment (MCI) [5], which is a prodromal form of Alzheimer’s disease (AD) [6]. Currently, East Africa has the highest number of people living with dementia in SSA (690,000 people) and is projected to have the highest proportionate increase in the number of people living with dementia (72%) between 2015 and 2030 [7]. In a survey conducted in Ugandan rural communities, one in five people aged 60 or older was found to have a probable diagnosis of dementia [8]. Although dementia is predominantly a disease among the elderly aged 65 years or older, there is increasing evidence of early onset dementia in people younger than 65 years [9, 10]. TBI is potentially one of the risk factors for early onset of dementia in young people.

Multiple pathological processes link TBI to neurodegeneration and dementia [11]. These include long-term brain changes and accumulation of pathological biomarkers in people with a history of TBI [12]. However, these findings are not consistent across persons with a history of TBI [13, 14] and some studies have not reported this association between TBI and dementia [15–17]. This controversy has been related to methodological approaches and failure to use standardized criteria to define both TBI and dementia [12, 17]. The other possible limitations of previous facility-based studies include reliance on patients admitted to hospital for ascertainment of dementia, scarce information on TBI severity, absence of comparison to non-TBI controls, and absence of death data [18]. Currently, there is limited evidence of this association and the influence of any other factors that exist in low- and middle-income countries where 60% of people with dementia live [19]. Previously, a study done in Central Uganda following up patients admitted with TBI reported the incidence of neurocognitive impairment to be 28.4% [20], although this study only followed up patients for 6 months and had a high loss to follow-up.

Traumatic brain injury (TBI) can have devastating life-long consequences that significantly reduce quality of life [21]. Recent improvements in critical care and rehabilitation have increased survival beyond the acute period, while the effects of TBI are carried across the lifespan as the people develop and grow older [22]. This may potentially increase the incidence of dementia in the aging population. Although some studies have investigated risk factors for dementia in the African population [23–27], there is paucity of information on TBI as a risk factor for dementia in the Ugandan population. The aim of this study was to determine the prevalence and factors associated with cognitive impairment following TBI in southwestern Uganda.

## Methods

### Study design and setting

This was a community-based cross-sectional study done in southwestern Uganda in May and June 2022. We used the Montreal Cognitive Assessment (MoCA) tool to screen for cognitive impairment among persons with a history of TBI. The study was conducted in nine selected communities of southwestern Uganda, purposively chosen due to the documented high prevalence of TBI.

### Study population and sample size

The study population included persons 18 years or older with a history of TBI in south-western Uganda. Only persons that had been discharged from hospital were included. Additionally, all participants must have experienced a TBI more than one month prior to the time of data collection. All participants gave written informed consent prior to inclusion in the study. Persons with visual disabilities were excluded from the study because the MoCA required visual ability. Persons who had had less than 10 years of education were screened with MoCA-B. The survey sample size was determined using the method from Hazra and Gogtay [28]. The Response Distribution (RD) was 0.28 based on previous findings that reported a prevalence of 28% of cognitive impairment following TBI in Uganda [20]. The margin of error (ME) used was 0.05, and a z-value of 1.96 was used. The formula for finite population correction was then applied to calculate the corrected sample size, with a population size of 579 based on the records of the TBI trauma register at Mbarara Regional Referral Hospital (MRRH). The calculated sample size was 202 respondents. We were able to recruit 189 participants in this study.

### Sampling criteria

The head trauma registers at MRRH were used to identify persons that met the study inclusion criteria. A member of the neurosurgery team that compiled the trauma register contacted the study participants for their availability to participate in the study. Participants that accepted to take part in the study were then followed up in the communities for recruitment. Additionally, district health educators were contacted to liaise with village health teams (VHT) to identify persons in the different communities with a history of head injury, and met the inclusion criteria for the study. Majority of the participants (152) were recruited through the VHTs, whereas only 37 participants could be traced from the hospital registry. This was due to the fact that most of the contacts in the hospital registry, that met the eligibility criteria for this study, either could not be reached or had passed on. Prior to enrolment, participants were interviewed for events surrounding the TBI and their reports were correlated with the report from the VHTs to avoid participant bias. Persons that consented to taking part in the study were invited to the nearest district health centre for screening. Four district health centres were chosen based on proximity to the study participants.

### Data collection tools

#### Questionnaire

Sociodemographic information was collected from the participants (or caretakers) including: gender (male or female), age (in years), level of education (no formal education, primary, secondary, undergraduate levels), history of smoking (yes or no) and history of alcohol use (yes or no). This study defined TBI as any penetrating and non-penetrating blunt and blast injuries to the brain. Information regarding the history of TBI was obtained for example; time since the last TBI (in months), number of TBIs experienced by the person, severity of the TBI (including the experience of any loss of consciousness), presence of any neurologic illness before the first TBI (such as clinically diagnosed dementia, CNS tumours, Parkinson’s), care received for the TBI (including length of hospital stay and medications or surgery) and the presence of any comorbidities in the patients (including but not limited to: HIV, hypertension, diabetes and any central nervous system—CNS disorders).

### Montreal Cognitive Assessment (MoCA)

The Montreal Cognitive Assessment (MoCA) is a standardized screening tool that was used to determine the presence of cognitive impairment in the study participants [29]. The MoCA was designed as a rapid screening instrument for mild cognitive dysfunction, and assesses different cognitive domains: attention and concentration, executive functions, memory, language, visuo-constructional skills, conceptual thinking, calculations, and orientation [30]. The MoCA was chosen because it is more sensitive than other tools in detection of cognitive impairment [31]. Importantly, the MoCA has previously been used and validated in a rural African setting in Tanzania [32] which is similar to our study setting. For participants that had more than 12 years in school of formal education, the MoCA tool was administered because literacy-dependent tasks may affect one’s score. Formal education in this study was defined as the structured education system in Uganda running from primary through secondary and tertiary levels. The MoCA was translated to Runyankole, the local dialect in southwestern Uganda and reviewed by a trained psychiatrist who was part of the research team and had a good understanding of Runyankole and Rukiga. In the naming section, the horse, tiger and duck were changed to a cow, lion and hen in order to provide pictures relevant to the local population. The total possible score on the MoCA was 30 points and a score of 26 or above was considered normal; a score of 19–24 was considered mild cognitive impairment; a score of 10 to 18 was considered moderate cognitive impairment and a score of less than 10 was considered sever cognitive impairment [29].

### Montreal Cognitive Assessment-Basic (MoCA-B)

The Montreal Cognitive Assessment -Basic (MoCA-B) was also adopted. MoCA-B is similar to the MoCA but was specifically created for screening patients with low education. Specifically, literacy-dependent tasks are eliminated and literacy-independent tasks that measured the same cognitive function are substituted in the MoCA-B. The MoCA-B was also translated to Runyankole to guide the research assistants in screening the participants. The total possible score on the MoCA-B was 30 points and a score of 26 or above was considered normal [33].

### Data collection procedures

Following ethics approval and administrative clearances from Mbarara University of Science and Technology Research Ethics Committee (MUST-2021-326) and MRRH Hospital Director respectively, the trauma register at MRRH was accessed to identify target participants. The study was also approved by the Uganda National Council for Science and Technology (HS2257ES). Contact details were extracted from the registers by the trauma team that had initially collected the data from the TBI patients. In cases where the target participant details were missing, those of the recorded caretaker were used. District Health Officers (DHOs) were also contacted for further administrative approval to contact persons with a history of TBI in the respective communities. Subsequently, district health coordinators and VHTs were contacted to identify persons in the different communities that had a history of head trauma and met the selection criteria for the study.

Identified persons with TBI history were briefed on the study and what it entailed. Those that accepted to take part in the study were then asked to report to the nearest health centre on a selected day for the screening to be done. The study was conducted by qualified research assistants with a background training (undergraduate or diploma) in a healthcare-related field. The four research assistants were trained on use of the study tools prior to data collection. The research assistants received a training based on the training and certification programme on the MoCA website (https://www.mocatest.org/get-certified/).

Prior to participating in the study, the research assistants took the potential participants through the consenting process, and all of the participants provided written informed consent. If the study participant met any of the criteria for determining a lack of capacity [34], like in cases of severe cognitive impairment, consent was received only from the caretakers or next of kin. However, in this study, no caretakers were required to provide consent. The sociodemographic questionnaire and the MoCA were then administered to the participant. The data collection process took approximately 25 minutes. An incentive in terms of transportation reimbursement depending on the distance travelled was provided to all study participants.

### Data management and analysis

In the field, data collection was done using printed questionnaires. Questionnaires were then reviewed for completeness at the end of each data collection day by the principal investigator. Completed questionnaires were kept in a locked cabinet and were only accessible to the lead author. In Kobo Toolbox, a data entry screen with checks was created before data being entered. Data was then transferred into the data analysis software, RStudio version 4.1.0. Data cleaning was done prior to the analysis. Continuous variables were analyzed using descriptive statistics that are: frequencies, means, medians and ranges and categorical variables using proportions, the T-test and chi-square analysis. Logistic regression was then done; first, bivariate analysis and then multivariate level adjusting for all variables. For the logistic regression model, some variables were recoded. The MoCA score was adjusted to a binary outcome, with a score greater than 25 being considered normal. Additionally, level of education was also adjusted to a binary outcome with participants that had gone beyond primary school being described as having eight or more years of formal education. Analysis was done at a 5% level of significance and only variables with a p-value less than 0.05 in the univariate regression were included in the final model. The primary outcome in this study was the presence of cognitive impairment, depending on the MoCA scores.

## Results

### Characteristics of study participants

**Fig 1:**
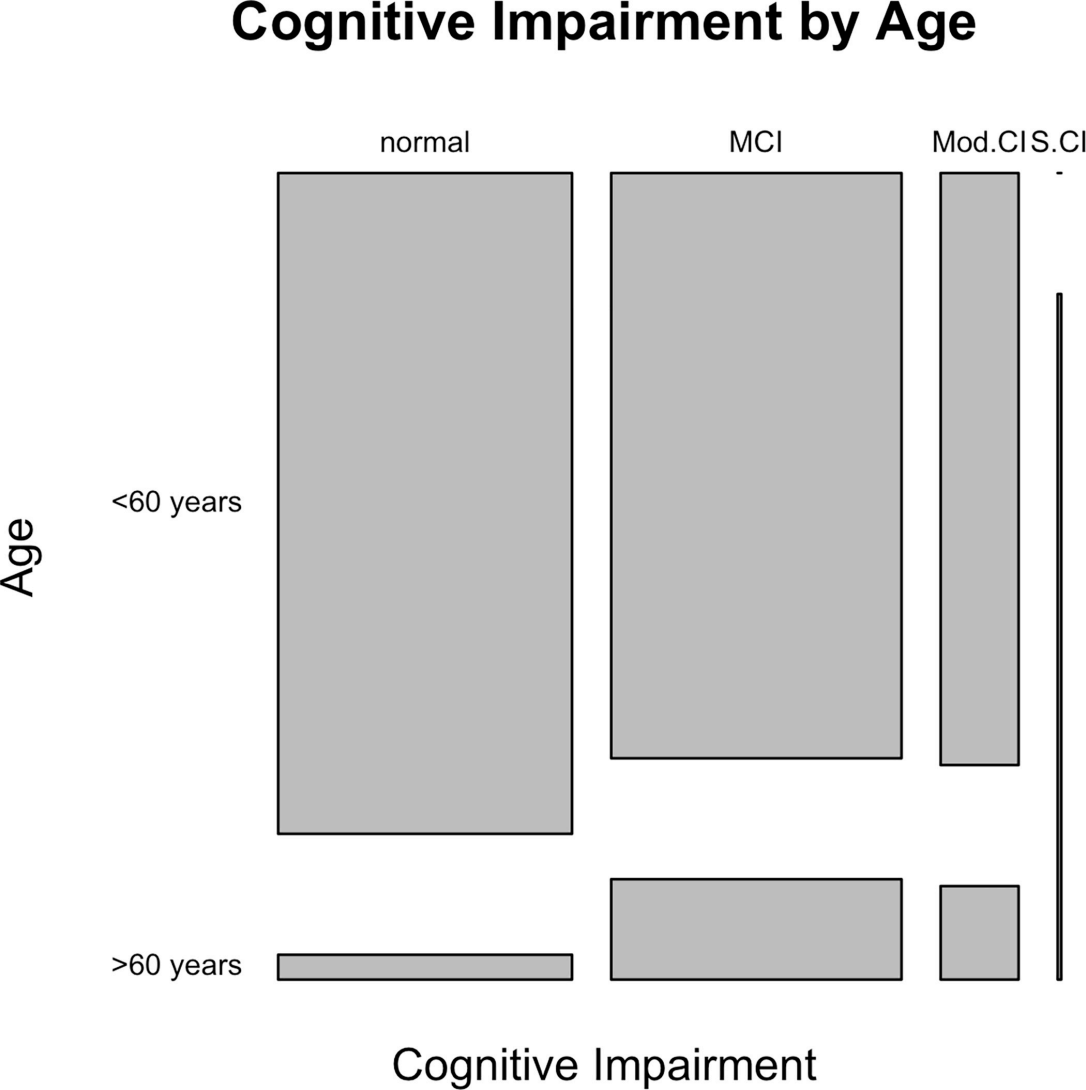
A mosaic plot of cognitive function by age. MCI—mild Cognitive Impairment; Mod. CI—Moderate Cognitive Impairment; S.CI—Severe Cognitive Impairment.

We enrolled 189 persons with a history of TBI in south-western Uganda. It was found that the majority of these persons (56.1%) had some form of cognitive impairment (**Table 1**). **Fig 1** shows a breakdown of cognitive impairment by age. The mean age of the study participants was 43.13±14.86 and majority of the study participants were male (78.7%). There was history of alcohol use among 137 participants (73.3%) and history of tobacco use among 49 participants (25.9%). Participants that had experienced a chronic deficit (such as loss of motor or sensory function or deficit in any of the special senses) following the TBI constituted 40.8% of the total population (**Table 1**).

**Table 1:** Baseline characteristics of the study participants.

Characteristic	Description	Total (%)
**Age**	Mean (sd)	43.13 (14.86)
**Gender**	Female	40 (21.3)
	Male	148 (78.7)
**Cognitive function**	Normal	83 (43.9)
	Mild Cognitive impairment	81 (43.1)
	Moderate cognitive impairment	22 (11.7)
	Severe cognitive impairment	2 (1.1)
**Level of Education**	No formal education	25 (13.2)
	Primary	115 (60.8)
	Secondary (A level)	11 (5.8)
	Secondary (O level)	34 (18.0)
	Undergraduate	4 (2.1)
**Number of TBIs**	More than one	37 (19.6)
	One	152 (80.4)
**Number of years since TBI**	Median (IQR)	3.0 (1.0 to 10.0)
**History of chronic disease**	No	160 (85.1)
	Yes	28 (14.9)
**History of alcohol use**	No history	50 (26.7)
	History	137 (73.3)
**Loss of consciousness following TBI**	No	27 (14.4)
	Yes	161 (85.6)
**History of tobacco use**	No history	140 (74.1)
	History	49 (25.9)
**Chronic deficit following TBI**	No	109 (59.2)
	Yes	75 (40.8)

### Factors associated with cognitive impairment in persons with a history of TBI

Age was identified to have an association with cognitive function; persons with cognitive impairment had a higher mean age as compared to those with normal cognitive function (47.2 vs. 38.0 years; *p*-value < 0.001). Loss of consciousness following the TBI (*p-*value 0.019) and a history of tobacco use were also associated with cognitive impairment among persons with a history of TBI (**Table 2**).

**Table 2:** Results from chi-square analysis and T-test showing association between the participants’ characteristics and cognitive impairment.

Characteristic	Description	Cognitive function		
		Normal	Cognitive Impairment	*p*-value
Total N = 189 (%)		83 (43.9)	106 (56.1)	
**Age**	Mean (SD)	38.0 (12.3)	47.2 (15.5)	**<0.01**
**Gender **	female	16 (19.3)	24 (22.9)	0.68
	male	67 (80.7)	81 (77.1)	
**Level of education**	8 years or more of formal education	23 (27.7)	26 (24.5)	0.743
	Less than 8 years of education	60 (72.3)	80 (75.5)	
**Number of TBIs **	more than one	18 (21.7)	19 (17.9)	0.62
	one	65 (78.3)	87 (82.1)	
**Number of years since TBI**	Mean (SD)	8.4 (11.2)	8.0 (11.8)	0.82
**History of chronic disease **	no	67 (80.7)	93 (88.6)	0.20
	yes	16 (19.3)	12 (11.4)	
**History of alcohol use **	no history	21 (25.6)	29 (27.6)	0.89
	history	61 (74.4)	76 (72.4)	
**Loss of consciousness following TBI **	no	18 (21.7)	9 (8.6)	**0.02 **
	yes	65 (78.3)	96 (91.4)	
**History of tobacco use **	no history	71 (85.5)	69 (65.1)	**<0.01 **
	history	12 (14.5)	37 (34.9)	
**Chronic deficit following TBI **	no	54 (67.5)	55 (52.9)	0.06
	yes	26 (32.5)	49 (47.1)	

### Predictors of cognitive impairment among persons with a history of TBI

Table 3 shows the results of a bivariate and multivariate logistic regression done to identify predictors of cognitive impairment among persons with a history of TBI. Notably, on multivariate adjustment, age (aOR = 1.04; CI = 1.01–1.07; *p*<0.01) and loss of consciousness following the TBI (aOR = 4.09; CI = 1.57–11.76; *p*<0.01) still remained significant predictors of having cognitive impairment in this cohort of patients (**Table 3**).

**Table 3:** Predictors of cognitive impairment among persons with a history of TBI.

Characteristic	Description	crude Odds Ratio (cOR)	*p-*value	adjusted Odds Ratio (aOR)	*p-*value
**Age**	**Mean (SD)**	**1.05 (1.03–1.08)**	**<0.01**	**1.04 (1.01–1.07)**	**<0.01**
**Gender**	Female	-		-	-
	Male	0.81 (0.39–1.63)	0.55	-	-
**Level of education**	8 or more years of formal education	-		-	-
	Less than 8 years	1.18 (0.61–2.27)	0.62	-	-
**Number of TBIs**	One	-		-	-
	More than one	0.79 (0.38–1.63)	0.52	-	-
**Number of years since TBI**	Mean (SD)	1.00 (0.97–1.02)	0.82	-	-
**History of chronic disease**	No	-		-	-
	Yes	0.49 (0.21–1.12)	0.1	**-**	**-**
**History of alcohol use**	No	-		-	-
	**Yes**	0.90 (0.46–1.73)	0.76	-	-
**Loss of consciousness following TBI**	No	-		-	
	**Yes**	**2.95 (1.28–7.27)**	**0.01**	**4.09 (1.57–11.76)**	**<0.01**
**History of tobacco use**	No	-		-	
	Yes	**3.17 (1.57–6.82)**	**<0.01**	2.01 (0.89–4.75)	0.1
**Chronic deficit following TBI**	No	-		-	
	Yes	**1.85 (1.02–3.42)**	**0.047**	1.54 (0.79–3.01)	0.21

## Discussion

The main objective of this study was to determine the prevalence and predictors of cognitive impairment among persons with a history of TBI in southwestern Uganda. The prevalence of cognitive impairment among this sub-population of patients was 56.1%, with age and loss of consciousness following the TBI identified as predictors of cognitive impairment.

The prevalence (56.1%) of cognitive impairment in our study is quite high. This study’s prevalence of cognitive impairment is slightly higher to that of a meta-analysis conducted by Tsai and colleagues in 2021 that estimated the prevalence of cognitive impairment to range between 20% and 50% [35]. Another study reported that following moderate to severe TBI, approximately 65% of patients report some form of impaired cognitive function [36], but the study measured cognitive function in terms of independence as opposed to memory, language, and communication domains utilized in our research. Additionally, none of these studies reported findings from an African setting. The prevalence is higher than that identified in a previous study done in the same setting that also used the MoCA to screen for dementia in elderly patients [37], possibly due to the fact that screening in the current study was done among individuals that already had an increased risk for cognitive impairment–TBI. Additionally, the MoCA has not been validated in our setting.

Of the study participants, 43% had mild cognitive impairment (MCI), 11.7% had moderate cognitive impairment and 1.1% had severe cognitive impairment. The high number of persons with mild cognitive impairment in this population is worrying given that it may be a prodromal form of Alzheimer’s disease (AD) [6]. Additionally, the likelihood of progression to any form of dementia is suggested to occur at a rate of 3 to 5 times higher in people with impaired cognition as compared to those with normal cognition [38]. This underscores the need to identify persons with MCI in this population and address modifiable risk factors that may hasten the progression to AD.

As a determinant of severity of TBI in this study, a self-report of loss of consciousness following the injury was assessed. Previously, loss of consciousness has been linked to severity of head injury [39, 40]. Our findings indicated that head injury leading to loss of consciousness had a four-fold risk of cognitive impairment. A previous study reported that individuals who experienced loss of consciousness following the TBI were at approximately 50% increased risk of dementia [41] while another reported a four-fold risk for dementia in head injury with loss of consciousness as compared to a two-fold risk for head injury without loss of consciousness [42]. Due to the fact that none of these studies reflected data from an African population, the present findings are important in guiding treatment interventions in patients in sub-Saharan Africa. Closer monitoring and long-term follow-up programs to give timely support, in respect to dementia development and progression, for TBI patients with history of loss of consciousness is necessary. Level of consciousness is an important indicator of TBI severity [43] especially in low-resource settings such as Uganda where it may not be possible to do diagnostic tests and detailed patient assessments. However, in this study, loss of consciousness was based on a self-report by study participants introducing a high likelihood of recall bias among the study participants. Additionally, a previous study in the US reported that use of level of consciousness as a proxy for TBI severity may be inaccurate due to use of sedatives [44]; that a participant in this study may have erroneously recalled as losing consciousness.

Previously, a dose response relationship has been described; with a mild single TBI showing a weaker association with dementia diagnosis than severe and more TBIs [45]. This has been attributed to the index TBI conferring vulnerability to cognitive outcomes in subsequent TBIs [46]. However, in this study only severity of the TBI was found to be a significant predictor of cognitive impairment and not the number of TBIs. This could be due to the low proportion of people (19.6%) that experienced multiple TBIs in this study. However, a study done in Sweden suggested that people that experience multiple TBIs may have had other risk factors for dementia such as low cognitive function and use of intoxicants prior to the TBI [47].

Age has been the most commonly identified risk factor for dementia [48] and among our study participants, age was an independent predictor for cognitive impairment. Current improvements in critical care and rehabilitation translate into improved survival of many TBI patients; carrying the effects of their injury across their lifespan as they develop and grow old [22]. This signifies the need for development of closer follow-up for TBI patients beyond acute care to enable timely identification and management of cognitive impairment, giving more attention to those with advanced age.

To our knowledge, this is among the first studies to document long-term cognitive impairment and risk factors among persons who have experienced TBI in sub-Saharan Africa. This presents an opportunity for future studies to build evidence base on modifiable risk factors for AD in people that experience TBI. However, one major limitation of this study is that it was based on patient self-report of TBI and the surrounding events such as loss of consciousness and thereby introducing recall bias and participant bias. Additionally, the TBI questionnaire was delivered prior to the MoCA, which could have introduced possible observer bias. Nonetheless, we correlated the self-report of TBI with that of community health workers to ensure accuracy of the history of TBI. The cohort of patients with a history of TBI may not have been large enough for our results to be generalizable. We suggest the use of larger trauma databases of persons with a history of TBI to confirm some of the associations identified in this study.

Given that this was a cross-sectional study, we could not establish a causal relationship between TBI and cognitive impairment. Therefore, we recommend cohort studies to follow up patients with TBI and establish the risk of development of cognitive impairment and subsequently, dementia. Although the MoCA has previously been used in southwestern Uganda [37], no formal tool validation has been done. However, the translated version of the MoCA was reviewed by a team of health professionals led by a consultant psychiatrist who had a good understanding of the Runyankore-Rukiga language. The lack of formal tool validation could have affected the results in this study. There is need for formal tool validation in this study setting. Finally, we did not establish a control group to determine the prevalence of cognitive impairment in the general population in Uganda. Subsequent cross-sectional studies should consider using non-TBI persons as controls, as this has been reported to reduce the effect of confounders that are likely to exist across both groups and may increase TBI-prone individuals’ risk for dementia [49], for instance, history of chronic diseases or history of alcohol use in this case.

## Conclusion and recommendations

There is a high burden of cognitive impairment among adults with a history of TBI in Uganda, confirming that TBI seems to have a role to play in the development of Cognitive Impairment even in our setting. There is a need to establish clinical management protocols that implement long-term follow-up of patients that experience TBI to enable early diagnosis, rehabilitation, and clinical management to slow progression to dementia. This study identified modifiable risk factors such as smoking as predictors of cognitive impairment. Behavioral modification programs among TBI patients should be provided, for instance, restriction of tobacco use; dietary and lifestyle interventions. Interestingly, alcohol use was found to be protective against the development of cognitive impairment. Longitudinal studies in the Ugandan population are needed to further explore the association between alcohol use and dementia.

## Supporting information

S1 Checklist STROBE guidelines for reporting observational studies. These are the guidelines that were followed in writing this manuscript.
(DOC)

S1 Data Document containing de-identified raw data on the study participants. (PDF)
