# Supplementary material for: Cognitive impairment following traumatic brain injury in Uganda: Prevalence and associated factors
Source: PLOS Glob Public Health. 2023 Feb 6;3(2):e0001459. doi: 10.1371/journal.pgph.0001459 (PMC10021383; doi:10.1371/journal.pgph.0001459)
Supplement: S1 Data — (PDF) [file pgph.0001459.s002.pdf]

Participant	age	gender	marital_sta	religion	tribe	occupatior	education	alcohol use
26	24	male	married	anglican	mukiga	unemploye	secondary (	ever used al
27	20	male	single	anglican	munyankol	businessma	secondary (	ever used al
28	44	female	married	anglican	munyankol	peasant	primary	never used
29	52	male	married	anglican	mukiga	peasant	primary	ever used al
30	38	male	married	catholic	munyankol	businessma	primary	Currently u
31	37	male	married	anglican	munyankol	unemploye	primary	Currently u
32	20	male	single	pentecosta	munyankol	other	secondary (	ever used al
32	33	male	married	catholic	munyankol	other	secondary (	ever used al
33	32	male	married	anglican	munyankol	unemploye	secondary (	never used
157	30	male	married	catholic	munyankol	bodaboda_	primary	Currently u
13	31	male	married	anglican	munyankol	other	primary	never used
8	31	male	married	muslim	munyankol	other	secondary (	never used
15	53	male	married	anglican	munyankol	peasant	secondary (	ever used al
7	43	male	married	catholic	munyankol	peasant	primary	ever used al
9	49	male	married	anglican	mukiga	peasant	secondary (	Currently u
11	49	male	married	anglican	munyankol	other	bachelor's (	never used
12	42	male	married	anglican	munyankol	businessma	primary	ever used al
16	39	male	married	catholic	mukiga	other	secondary (	never used
25	18	male	single	catholic	munyankol	unemploye	primary	never used
24	25	male	single	pentecosta	munyankol	bodaboda_	primary	Currently u
149	48	male	married	catholic	munyankol	other	primary	ever used al
151	30	male	married	anglican	munyankol	peasant	secondary (	Currently u
152	48	male	married	pentecosta	munyankol	peasant	primary	ever used al
153	72	male	separated	anglican	munyankol	peasant	primary	Currently u
120	43	male	married	other	munyankol	peasant	primary	ever used al
118	54	male	married	catholic	munyankol	unemploye	primary	Currently u
180	38	female	married	catholic	munyankol	peasant	primary	never used
144	47	female	separated	catholic	munyankol	peasant	secondary (	ever used al
105	49	female	married	anglican	munyankol	peasant	primary	never used
111	67	male	separated	catholic	munyankol	other	primary	never used
108	38	male	married	catholic	munyankol	peasant	secondary (	ever used al
100	67	male	married	pentecosta	munyankol	other	primary	ever used al
102	65	male	married	catholic	munyankol	peasant	primary	Currently u
23	42	female	married	anglican	munyankol	peasant	primary	Currently u
22	22	male	married	catholic	mukiga	unemploye	primary	Currently u
192	73	female	separated	pentecosta	mukiga	peasant	no formal e	ever used al
191	64	female	separated	anglican	munyankol	peasant	no formal e	ever used al
193	54	female	married	anglican	mukiga	other	secondary (	never used
190	65	female	separated	catholic	munyankol	other	secondary (	ever used al
189	52	female	separated	catholic	munyankol	peasant	primary	never used
195	103	male	married	anglican	munyankol	peasant	no formal e	ever used al
188	42	male	married	catholic	munyankol	peasant	primary	Currently u
187	40	male	married	anglican	mukiga	unemploye	primary	ever used al
186	59		separated	anglican	munyankol	peasant	no formal e	ever used al
185	58	male	married	other	munyankol	peasant	no formal e	never used
184	28	male	married	pentecosta	munyankol	other	primary	ever used al
36	47	male	married	pentecosta	munyankol	businessma	primary	Currently u
35	66	male	married	pentecosta	munyankol	unemploye	no formal e	ever used al
34	25	male	single	anglican	munyankol	other	bachelor's (	ever used al
148	37	male	married	anglican	munyankol	other	primary	Currently u
140	63	male	married	catholic	munyankol	peasant	secondary (	ever used al

139	60	female	separated	anglican	munyankol	peasant	primary	never used
2	53	female	married	anglican	muganda	peasant	primary	never used
116	96	male	married	anglican	munyankol	other	primary	ever used al
3	51	male	married	catholic	munyankol	peasant	primary	
113	54	male	married	other	munyankol	peasant	primary	ever used al
122	29	female	married	catholic	munyankol	peasant	primary	never used
1	61	male	married	anglican	muganda	businessma	primary	ever used al
125	58	female	married	anglican	munyankol	peasant	primary	ever used al
137	47	male	married	catholic	munyankol	peasant	primary	Currently u
136	39	male	married	muslim	munyankol	bodaboda_	primary	never used
126	24	male	married	catholic	munyankol	other	secondary (	never used
4		male	separated	anglican	munyankol	peasant	primary	ever used al
6	17	male	single	catholic	munyankol	other	secondary (	never used
128	63	female	separated	anglican	munyankol	peasant	primary	ever used al
130	61	female	married	catholic	munyankol	peasant	primary	ever used al
132	44	female	separated	catholic	munyankol	businessma	primary	never used
5	23	male	single	pentecosta	other	businessma	secondary (	ever used al
14	20	male	single	anglican	munyankol	peasant	primary	never used
21	40	male	married	anglican	munyankol	other	primary	ever used al
20	21	male	single	catholic	munyankol	other	primary	ever used al
19	35	male	married	catholic	munyankol	unemploye	primary	Currently u
18	38	male	married	pentecosta	mukiga	bodaboda_	secondary (	ever used al
17	33	male	married	catholic	munyankol	other	primary	ever used al
16	30	male	single	catholic	munyankol	unemploye	primary	Currently u
7	52	male	married	catholic	munyankol	unemploye	primary	Currently u
8	63	male	married	pentecosta	munyankol	other	no formal e	ever used al
9	54	female	separated	catholic	munyankol	peasant	no formal e	ever used al
10	26	male	married	anglican	munyankol	businessma	primary	never used
11	27	male	married	anglican	munyankol	other	secondary (	ever used al
12	24	male	married	catholic	munyankol	other	primary	Currently u
13	39	male	married	pentecosta	munyankol	bodaboda_	no formal e	ever used al
14	25	male	single	catholic	munyankol	businessma	secondary (	ever used al
15	34	male	married	catholic	mukiga	other	primary	Last used le
155	51	male		anglican	munyankol	peasant	no formal e	Currently u
154	60	female	separated	catholic	munyankol	peasant	primary	ever used al
156	37	male	married	catholic	munyankol	bodaboda_	primary	Currently u
38	47	female	married	anglican	munyankol	peasant	primary	never used
39	58	male	married	anglican	munyankol	peasant	secondary (	Currently u
40	25	male	married	anglican	munyankol	bodaboda_	primary	Currently u
41	60	female	married	catholic	munyankol	peasant	no formal e	ever used al
42	53	male	married	anglican	munyankol	peasant	primary	Currently u
43	28	male	married	anglican	munyankol	bodaboda_	secondary (	ever used al
44	42	male	married	catholic	munyankol	peasant	primary	Currently u
45	34	male	married	catholic	munyankol	peasant	primary	Currently u
46	44	male	married	anglican	munyankol	businessma	primary	never used
47	40	male	married	anglican	munyankol	businessma	bachelor's (	Currently u
48	41	male	married	anglican	munyankol	peasant	secondary (	ever used al
146	25	female	married	catholic	munyankol	peasant	primary	never used
182	47	male	married	anglican	mukiga	peasant	secondary (	Currently u
182	50	male	married	anglican	munyankol	other	secondary (	never used
196	77	male	married	anglican	munyankol	peasant	secondary (	ever used al

194	42	male	married	anglican	munyankol	other	primary	Currently u
158	37	female	married	catholic	mukiga	unemploye	primary	never used
37	51	male	married	pentecosta	munyankol	peasant	no formal e	ever used al
160	51	male	married	catholic	munyankol	other	bachelor's (	Currently u
104	49	female	married	anglican	munyankol	peasant	primary	never used
162	51	male	married	anglican	munyankol	peasant	no formal e	Last used le
161	53	male	married	anglican	munyankol	businessma	primary	Currently u
163	50	female	married	catholic	munyankol	peasant	no formal e	ever used al
165	59	female	married	catholic	munyankol	peasant	no formal e	never used
165	34	male	married	anglican	munyankol	other	primary	Currently u
166	51	male	married	anglican	munyankol	peasant	primary	Last used le
168	26	female	separated	anglican	munyankol	peasant	primary	Currently u
169	42	male	married	catholic	munyankol	peasant	primary	ever used al
167	56	male	married	anglican	munyankol	other	secondary (	ever used al
27	36	male	single	anglican	munyankol	bodaboda_	primary	never used
17	29	male	married	anglican	munyankol	other	primary	never used
20	32	male	married	catholic	munyankol	other	secondary (	Currently u
19	50	male	married	anglican	munyankol	other	primary	Currently u
18	50	male	married	catholic	munyankol	bodaboda_	primary	Currently u
21	26	male	married	anglican	munyankol	bodaboda_	secondary (	ever used al
22	34	male	married	anglican	munyankol	bodaboda_	primary	Currently u
23	34	male	married	catholic	other	other	primary	Currently u
24	28	male	married	anglican	munyankol	bodaboda_	primary	Currently u
25	42	male	married	catholic	munyankol	bodaboda_	primary	ever used al
141	56	female	separated	anglican	munyankol	peasant	no formal e	ever used al
28	4	male	married	anglican	munyankol	bodaboda_	primary	Last used le
26	28	male	single	anglican	munyankol	bodaboda_	primary	ever used al
30	52	male	married	anglican	munyankol	other	primary	Last used le
31	50	male	married	pentecosta	munyankol	peasant	primary	ever used al
29	26	male	single	anglican	munyankol	bodaboda_	secondary (	Last used le
107	27	female	married	catholic	munyankol	peasant	primary	never used
101	63	male	married	anglican	munyankol	other	secondary (	ever used al
121	56	male	married	catholic	munyankol	peasant	primary	ever used al
124	58	male	married	catholic	munyankol	peasant	primary	ever used al
123	51	female	married	catholic	munyankol	peasant	primary	never used
127	31	female	married	catholic	munyankol	businessma	primary	never used
129	26	male	single	catholic	munyankol	peasant	primary	never used
133	40	male	married	catholic	munyankol	bodaboda_	primary	Currently u
143	3	male	married	catholic	munyankol	bodaboda_	secondary (	never used
142	65	male	married	catholic	munyankol	peasant	primary	ever used al
145	32	male	married	catholic	munyankol	unemploye	secondary (	never used
147	36	female	married	catholic	munyankol	businessma	secondary (	ever used al
112	40	male	married	anglican	munyankol	other	secondary (	Currently u
110	36	male	married	catholic	munyankol	other	secondary (	ever used al
102	50	female	separated	catholic	munyankol	peasant	no formal e	never used
114	31	male	married	catholic	munyankol	peasant	secondary (O level)	
115	55	female	married	catholic	munyankol	peasant	no formal e	ever used al
117	41	male	married	pentecosta	munyankol	unemploye	primary	Last used le
119	43	male	married	other	munyankol	businessma	primary	ever used al
13	40	female	separated	catholic	munyankol	peasant	primary	ever used al
138	52	male	married	catholic	munyankol	peasant	primary	ever used al
135	38	male	separated	catholic	munyankol	businessma	secondary (	ever used al

134	46	male	married	catholic	munyankol	other	primary	Currently u
608	49	male	married	other	munyankol	other	secondary (	never used
607	57	female	married	anglican	munyankol	peasant	primary	never used
606	45	male	married	muslim	mukiga	other	no formal e	never used
605	56	male	married	catholic	muganda	other	no formal e	Currently u
604	38	male	married	catholic	munyankol	unemploye	primary	Currently u
603	42	male	married	anglican	munyankol	peasant	no formal e	ever used al
602	53	male	married	anglican	mukiga	peasant	no formal e	Currently u
601	50	male	married	anglican	munyankol	other	primary	ever used al
600	40	male	single	anglican	munyankol	other	secondary (	Currently u
609	39	male	married	anglican	munyankol	other	secondary (	Currently u
714	25	male	single	pentecosta	mukiga	bodaboda_	primary	never used
713	45	male	single	catholic	munyankol	peasant	primary	ever used al
712	43	male	married	pentecosta	munyankol	peasant	no formal e	ever used al
613	39	male	married	other	munyankol	peasant	primary	never used
613	40	male	married	catholic	munyankol	other	primary	ever used al
611	23	male	single	anglican	munyankol	peasant	primary	never used
610	25	male	married	anglican	munyankol	bodaboda_	secondary (	never used
712	77	male	separated	anglican	munyankol	peasant	no formal e	Currently u
711	31	male	married	anglican	munyankol	bodaboda_	primary	ever used al
710	43	female	married	anglican	mukiga	businessma	primary	never used
709	43	male	single	anglican	munyankol	bodaboda_	primary	Last used le
708	47	male	single	anglican	munyankol	bodaboda_	primary	never used
707	40	female	single	pentecosta	munyankol	businessma	primary	never used
706	42	male	single	anglican	munyankol	peasant	primary	never used
705	41	male	married	anglican	munyankol	peasant	primary	ever used al
704	56	male	separated	anglican	munyankol	businessma	primary	Currently u
703	50	male	married	anglican	mukiga	peasant	no formal e	Currently u
702	46	female	single	catholic	munyankol	peasant	secondary (	Currently u
701	56	male	single	anglican	munyankol	businessma	secondary (	Last used le
700	56	male	married	catholic	munyankol	peasant	primary	Currently u
25	18	male	single	catholic	munyankol	unemploye	primary	never used
24	25	male	single	pentecosta	munyankol	bodaboda_	primary	Currently u
23	42	female	married	anglican	munyankol	peasant	primary	Currently u
22	22	male	married	catholic	mukiga	unemploye	primary	Currently u

tobacco us	use of othe	number of	age at most	age at first	Years that h	loss of cons	how long w	chronic def
never used	never used	1	23	23	0.5	yes	more than !	failure to m
never used	never used	1	18	18	1	yes	more than !	none
never used	never used	1	43	43	1	yes	more than !	loss of sens
ever used b	ever used d	1	49	49	1	yes	more than !	loss of any c
never used	never used	1	37	37	1	yes	more than !	loss of any c
never used	never used	1	34	34	3	yes	more than !	failure to m
never used	never used	1	18	18	2	yes	more than !	none
never used	never used	1	31	31	2	yes	more than !	failure to m
never used	never used	1	32	32	1	yes	more than !	none
never used	never used	1	28	28	2	no		none
never used	never used	1	29	29	1.5	yes	can't remer	none
never used	never used	1	31	31	0.42	yes	more than !	none
never used		1	44	44	4	yes	more than !	none
never used	never used	1	42	42	0.3	yes	can't remer	failure to m
never used	never used	1	48	48	0.3	yes	can't remer	none
never used	never used	1	48	48	1	yes	can't remer	loss of any c
never used	never used	1	40	40	2	yes	can't remer	failure to m
never used	never used	1	36	36	3	yes	more than !	none
never used	never used	1	16	16	2	yes	more than !	none
never used	never used	1	23	23	2	yes	more than !	none
ever used b	never used	1	25	25	23	yes	more than !	none
currently u	currently u	1	29	29	0.5	yes	more than !	none
never used	never used	1	45	45	3	yes	more than !	failure to m
ever used but more tha		1	72	72	0.5	no		none
never used	never used	1	43	43	0.17	yes	less than 5	loss of sens
never used	currently u	2	53	43	1	yes	can't remer	none
never used	never used	2	36	26	2	yes	more than 5 minutes	
never used	never used	2	24	13	23	yes	more than !	none
never used		1	45	45	4	no		none
currently u	never used	2	53	42	25	yes	more than !	loss of sens
never used	never used	1	38	38	0.167	yes	more than !	none
never used	never used	1	60	60	7	yes	more than !	loss of sens
ever used b	never used	1	60	60	5	yes	more than !	none
never used	never used	1	39	39	3	yes	more than !	loss of sens
never used	never used	1	20	20	2	yes	more than !	loss of any c
never used	never used	1	22	22	51	no		failure to m
ever used b	never used	2	63	40	1	yes	less than 5	none
never used	never used	2	14	7	40	yes	can't remer	none
ever used b	never used	1	51	51	14	yes	more than !	loss of sens
never used	never used	1	49	49	3	yes	more than !	none
ever used b	never used	1	83	83	20	no		none
ever used b	never used	1	41	41	1	yes	more than !	loss of sens
ever used b	never used	2	39	20	1	yes	more than !	none
currently u	never used	1	18	18	41	yes	more than !	none
never used	never used	1	23	23	35	yes	more than !	failure to m
never used	never used	2	24	23	4	no		loss of sens
ever used b	never used	1	32	32	4	yes	more than !	none
ever used b	never used	1	62	62	4	yes	more than !	none loss o
never used	never used	1	24	24	1	yes	more than !	loss of any c
never used	never used	1	19	19	18	yes	more than !	none
never used	never used	1	58	58	5	yes	less than 5	none

never used	never used	1	55	55	5	yes	more than	loss of any
never used	never used	1	52		11	yes	can't remember	
never used	never used	1	70	70	26	yes	can't remer	failure to m
ever used b	never used	2	5	10	0.3	yes	more than	none
never used	never used	1	50	50	4	yes	more than	loss of any
never used	never used	1	17	17	12	yes	more than	loss of any
never used	never used	1	60		0.54	yes	can't remer	none
ever used b	never used	2	53	50	5	yes	more than	loss of sens
never used	never used	1	22	32	25	yes	more than	none
never used		1	35	35	4	yes	less than 5	none
never used	never used	1	23	23	1	yes	more than 5 minutes	
currently u	never used	1	55		3	yes	can't remer	none
never used	never used	1	16	16	0.25	yes	can't remer	loss of sens
ever used b	never used	2	61	30		yes	less than 5	loss of sens
ever used b	never used	1	49	49	61	yes	less than 5	none
never used	never used	1	42	42	1	yes	more than	none
never used	never used	1	19	19	2.5	yes	can't remer	none
never used	never used	1	20	20	0.3	yes	more than	none
ever used b	never used	1	36	36	4	yes	more than	loss of sens
never used	never used	1	21	21	0.42	yes	more than	none
ever used b	ever used d	1	34	34	1	yes	more than	failure to m
never used	never used	1	36	36	3	yes	more than	none
never used	never used	2	32	30	1	yes	more than	loss of any
never used	never used	2	30	29	0.25	no		loss of any
ever used b	never used	1	50	50	2	yes	can't remer	failure to m
ever used b	never used	1	60	60	3	yes	more than	failure to m
never used	never used	1	50	50	4	yes	more than	loss of any
never used	never used	1	24	24	2	no		none
never used	never used	1	24	24	3	yes	more than	none
never used	never used	1	23	23	1	yes	more than	loss of any
never used	never used	1	26	26	3	yes	more than	none
never used	never used	1	25	25	0.5	yes	more than	loss of any
never used	never used	2	34	30	4	yes	more than	loss of sens
never used	never used	1	50	50	1	yes		none
ever used b	never used	1	52	52	8	yes	more than	none
never used	never used	1	23	23	14	yes	more than	none
never used	never used	1	37	37	10	yes	more than	none
ever used b	never used	1	57	57	1	no		none
never used	never used	2	22	20	2	yes	more than	none
currently u	never used	1	52	52	8	yes	more than	failure to m
ever used b	never used	2	40	37	2	yes	more than	none
ever used b	never used	1	28	28	0.42	yes	more than	none
never used	never used	1	16	16	28	yes	more than	none
currently u	never used	2	45	25	0.42	yes	more than	none
never used	never used	1	42	42	2	yes	more than	none
never used	never used	1	38	38	9	no		none
currently u	never used	1	37	37	4	yes	more than	none
never used	never used	1	23	23	2	no		none
never used	never used	1	11	11	36			none
never used	never used	2	37	17	13	yes	more than	failure to m
never used	never used	1	70	70	7	no		loss of any

currently u	never used	1	38	38	4	yes	more than !	loss of any c
never used	never used	1	36	36	0.6	yes	more than !	none
ever used b	never used	1	45	45	6	yes	more than !	failure to m
never used	never used	2	48	47	3	yes	more than !	none
never used	never used	1	44	44	5	yes	more than !	none
never used	never used	1	36	36	15	yes	more than !	none
ever used b	never used	1	52	52	1	yes	more than !	none
never used	never used	1	28	28	22	yes	more than !	loss of any c
never used	never used	1	49	49	10	yes	more than !	none
never used	never used	1	34	34	0.42	yes	more than !	none
last used le	never used	1	48	48	3	yes	more than !	none
never used	never used	1	12	12	14	yes	more than !	none
never used	never used	1	8	8	34	no		none
ever used b	never used	1	35	35	21	yes	more than !	none
never used	never used	1	33	33	3	yes	more than !	none
never used	never used	1	24	24	5	yes	more than !	none
never used	never used	1	22	22	10	no		none
ever used b	never used	1	13	13	37	yes	can't remer	none
never used	never used	1	33	33	7	yes	more than !	none
never used	never used	1	14	14	12	yes	more than !	none
never used	never used	1	29	29	4.5	no		none
never used	never used	1	20	20	14	no		none
never used	never used	1	27	27	1	yes	more than 5 minutes	
never used	never used	1	40	40	2	yes	more than !	none
never used	never used	1	5	5	51	yes	can't remer	none
never used	never used	2	39	20	1	yes	more than !	none
never used		1	27	27	1	no		none
never used	never used	1	12	12	40	no		none
ever used b	never used	2	32	24	18	no		none
never used	never used	1	15	15	11	yes	can't remer	none
never used	never used	1	11	11	11	yes	more than !	none
never used	never used	1	61	61	2	no		none
never used	never used	1	14	14	42	yes	less than 5 ı	loss of sens
ever used b	never used	1	26	26	32	yes	more than !	none
ever used b	never used	1	33	33	18	yes	less than 5 ı	loss of sens
never used	never used	1	14	14	18	yes	less than 5 ı	none
never used	never used	2	21	3	5	yes	less than 5 ı	none
currently u	never used	1	35	36	5	yes	more than !	none
never used	never used	1	22	22	9	yes	less than 5 ı	none
ever used b	never used	2	63	31	1	yes	more than !	loss of any c
never used	never used	2	32	8	24	yes	less than 5 ı	none
never used	never used	1	27	27	9	yes	less than 5 ı	failure to m
never used	never used	1	39	39	1	no		none
never used	never used	1	28	28	8	yes	more than !	none
never used	never used	1	44	44	6	yes	more than !	none
never used	never used	1	25	25	6	yes	can't remer	loss of any c
never used	ever used d	1	6	6	49	yes	can't remer	loss of sens
never used	never used	2	37	2	4	no		none
never used	never used	2	42	36	1	yes	more than !	loss of any c
never used	never used	1	32	32	8	yes	less than 5 ı	none
never used	never used	2	31	28	21	yes	less than 5 ı	loss of any c
never used	never used	1	25	25	12	yes	less than 5 ı	loss of any c

currently u	never used	2	36	30	10	yes	more than !	loss of sens:
never used	never used	1	40	40	8	yes	less than 5 ı	none
never used	never used	2	54	14	3	yes	more than !	failure to m
currently u	never used	1	43	43	4	no		none
currently u	never used	4	50	30	6	yes	more than !	none
never used	never used	1	37	37	1	yes	more than !	loss of sens:
never used	never used	2	40	33	2	yes	more than !	none
currently u	never used	3	52	25	0.58	yes	more than !	none
never used	never used	1	54	54	6	yes	more than !	none
never used		1	49	39	0.75	yes	can't remer	failure to m
currently u	never used	1	31	31	8	yes	more than !	none
never used	never used	1	23	23	1.6	yes	more than !	loss of sens:
never used	never used	1	39	39	6	yes	less than 5 ı	failure to m
never used	never used	2	41	30	4	yes	can't remer	loss of any (
never used	never used	1	37	37	2	no		
currently u	never used	1	20	20	20	no		none
never used	never used	2	19	13	3	yes	more than !	none
never used	never used	2	23	12	1.5	yes	more than !	none
currently u	never used	1	67	67	9	yes	can't remer	failure to m
never used	never used	1	30	30	1	yes	can't remer	failure to m
never used	never used	1	40	40	4	yes	can't remer	failure to m
never used	never used	1	39	39	4	yes	can't remer	loss of sens:
never used	never used	1	25	25	22	no		loss of sens:
never used	never used	1	40	40	0.33	yes	can't remer	failure to m
never used	never used	1	30	30	12	no		loss of any (
never used	never used	1	40	40	0.58	yes	can't remer	failure to m
ever used b	never used	2	55	10	0.5	yes	can't remer	failure to m
never used	never used	1	27	27	23	yes	can't remer	failure to m
never used	never used	1	43	43	3	yes	more than !	failure to m
never used	never used	1	56	56	0.25	yes	can't remer	none
never used	never used	2	56	36	1	yes	can't remer	loss of sens:
never used	never used	1	16	16	2	yes	more than !	none
never used	never used	1	23	23	2	yes	more than !	none
never used	never used	1	39	39	3	yes	more than !	loss of sens:
never used	never used	1	20	20	2	yes	more than !	loss of any (

chronic del	chronic del	chronic del	chronic del	manageme	treatment r	history of c	cns disorde	chronic illn
0	1	0	1	hospitalise	medication	no		none of the
1	0	0	0	hospitalise	surgery and	no		none of the
0	0	0	1	hospitalise	medication	no		none of the
0	0	1	1	hospitalise	surgery and	no		none of the
0	0	1	0	hospitalise	medication	no		none of the
0	1	0	0	hospitalise	medication	no		none of the
1	0	0	0	hospitalise	medication	no		none of the
0	1	0	1	hospitalise	medication	no		none of the
1	0	0	0	hospitalise	medication	no		none of the
1	0	0	0	hospitalise	medication	no		none of the
1	0	0	0	hospitalise	surgery and	no		none of the
1	0	0	0	hospitalise	surgery and	no		none of the
1	0	0	0	hospitalise	medication	no		none of the
0	1	0	0	hospitalise	surgery and	no		none of the
1	0	0	0	hospitalise	medication	no		none of the
0	0	1	0	hospitalise	medication	no		none of the
0	1	0	1	hospitalise	medication	no		none of the
1	0	0	0	hospitalise	medication	no		none of the
1	0	0	0	hospitalise	medication	no		none of the
1	0	0	0	hospitalise	medication	no		none of the
1	0	0	0	hospitalise	medication	no		HIV/AIDS
1	0	0	0	hospitalise	medication	no		none of the
0	1	0	0	hospitalise	medication	no		none of the
1	0	0	0	hospitalise	medication	no		none of the
0	0	0	1	hospitalise	medication	no		none of the
1	0	0	0	hospitalise	medication	no		none of the
				hospitalise	medication	no		none of the
1	0	0	0	hospitalise	medication	no		none of the
1	0	0	0	hospitalise	medication	no		
0	0	0	1	hospitalise	medication	no		none of the
1	0	0	0	hospitalise	medication	no		none of the
0	0	0	1	hospitalise	medication	no		HIV/AIDS
1	0	0	0	hospitalise	surgery and	no		hypertensio
0	0	0	1	hospitalise	medication	no		hypertensio
0	0	1	0	hospitalise	medication	no		none of the
0	1	0	0	hospitalise	medication	no		none of the
1	0	0	0	not hospita	medication	no		none of the
1	0	0	0	hospitalise	medication	no		none of the
0	0	0	1	hospitalise	medication	no		none of the
1	0	0	0	hospitalise	medication	no		none of the
1	0	0	0	not hospita	no treatme	no		none of the
0	0	0	1	hospitalise	medication	no		none of the
1	0	0	0	hospitalise	surgery and	no		HIV/AIDS
1	0	0	0	not hospita	medication	no		none of the
0	1	0	0	hospitalise	medication	no		none of the
0	0	0	1	hospitalise	medication	no		none of the
1	0	0	0	hospitalise	medication	no		none of the
1	0	0	1	hospitalise	surgery and	no		none of the
0	0	1	0	hospitalise	medication	no		none of the
1	0	0	0	hospitalise	medication	no		none of the
1	0	0	0	hospitalise	medication	no		none of the

0	0	1	0	hospitalise	medication	no		none of the
				hospitalise	medication	no		
0	1	0	0	hospitalise	medication	no		hypertensio
1	0	0	0	hospitalised		no		none of the
0	0	1	1	not hospita	medication	no		HIV/AIDS
0	0	1	0	hospitalise	medication	no		none of the
1	0	0	0	hospitalise	medication	no		none of the
0	0	0	1	hospitalise	medication	no		none of the
1	0	0	0	hospitalise	medication	no		none of the
1	0	0	0	hospitalise	medication	no		none of the
				hospitalise	medication	no		none of the
1	0	0	0	hospitalise	medication	no		none of the
0	0	0	1	hospitalise	medication	no		
0	0	0	1	hospitalise	medication	no		hypertensio
1	0	0	0	hospitalise	medication	no		none of the
1	0	0	0	hospitalise	medication	no		HIV/AIDS
1	0	0	0	hospitalise	medication	yes	other	none of the
1	0	0	0	hospitalise	medication	no		
0	0	0	1	hospitalise	medication	no		HIV/AIDS
1	0	0	0	hospitalise	medication	no		none of the
0	1	0	0	hospitalise	medication	no		none of the
1	0	0	0	hospitalise	surgery and	no		none of the
0	0	1	0	hospitalise	surgery and	no		none of the
0	0	1	0	hospitalise	surgery and	no		none of the
0	1	1	1	hospitalise	surgery and	no		none of the
0	1	1	1	hospitalise	surgery and	no		none of the
0	0	1	0	hospitalise	medication	no		none of the
1	0	0	0	hospitalise	medication	no		none of the
1	0	0	0	hospitalise	no treatme	no		none of the
0	0	1	0	hospitalise	medication	no		none of the
1	0	0	0	hospitalise	medication	no		HIV/AIDS
0	0	1	0	hospitalise	surgery and	no		none of the
0	0	0	1	hospitalise	medication	no		none of the
1	0	0	0	hospitalise	medication	no		none of the
1	0	0	0	hospitalise	medication	no		none of the
1	0	0	0	hospitalise	medication	no		none of the
1	0	0	0	hospitalise	medication	no		none of the
1	0	0	0	hospitalise	medication	no		none of the
1	0	0	0	hospitalise	medication	no		none of the
0	1	1	1	hospitalise	medication	no		none of the
1	0	0	0	hospitalise	medication	no		none of the
1	0	0	0	hospitalise	medication	no		none of the
1	0	0	0	not hospita	no treatme	no		HIV/AIDS
1	0	0	0	hospitalise	medication	no		none of the
1	0	0	0	hospitalise	medication	no		none of the
1	0	0	0	hospitalise	medication	no		none of the
1	0	0	0	hospitalise	medication	no		none of the
1	0	0	0	hospitalise	medication	no		none of the
1	0	0	0	hospitalise	medication	no		hypertensio
0	1	1	0	hospitalise	medication	no		none of the
0	0	1	0	hospitalise	medication	no		diabetes

0	0	1	0	hospitalise	medication	no	HIV/AIDS
1	0	0	0	hospitalise	medication	no	none of the
0	1	0	0	hospitalise	medication	no	none of the
1	0	0	0	hospitalise	medication	no	none of the
1	0	0	0	hospitalise	surgery and	no	none of the
1	0	0	0	hospitalise	surgery and	no	none of the
1	0	0	0	hospitalise	medication	no	none of the
0	0	1	0	hospitalise	medication	no	none of the
1	0	0	0	hospitalise	medication	no	none of the
1	0	0	0	hospitalise	medication	no	none of the
1	0	0	0	hospitalise	medication	no	HIV/AIDS
1	0	0	0	hospitalise	medication	no	HIV/AIDS
1	0	0	0	hospitalise	medication	no	none of the
1	0	0	0	hospitalise	surgery and	no	HIV/AIDS
1	0	0	0	hospitalise	medication	no	HIV/AIDS
1	0	0	0	hospitalise	medication	no	none of the
1	0	0	0	hospitalise	medication	no	none of the
1	0	0	0	hospitalise	medication	no	none of the
1	0	0	0	hospitalise	no treatme	no	none of the
1	0	0	0	hospitalise	medication	no	none of the
1	0	0	0	hospitalise	no treatme	no	none of the
1	0	0	0	hospitalise	medication	no	
				hospitalise	medication	no	none of the
1	0	0	0	hospitalise	surgery and	no	
1	0	0	0	hospitalise	medication	no	none of the
1	0	0	0	hospitalise	medication	no	none of the
1	0	0	0	hospitalise	medication	no	none of the
1	0	0	0	hospitalise	medication	no	hypertensio
1	0	0	0	not hospitalised		no	none of the
1	0	0	0	not hospita	no treatme	no	HIV/AIDS
1	0	0	0	hospitalise	medication	no	none of the
1	0	0	0	hospitalise	medication	no	none of the
0	0	0	1	hospitalise	medication	no	none of the
1	0	0	0	hospitalise	medication	no	none of the
0	0	0	1	hospitalise	medication	no	none of the
1	0	0	0	hospitalise	medication	no	none of the
1	0	0	0	hospitalise	medication	no	none of the
1	0	0	0	hospitalise	medication	no	HIV/AIDS
1	0	0	0	hospitalise	medication	no	none of the
0	0	1	0	hospitalise	medication	no	none of the
1	0	0	0	hospitalise	medication	no	none of the
0	1	0	0	hospitalise	medication	no	
1	0	0	0	hospitalise	surgery and	no	none of the
1	0	0	0	hospitalise	surgery and	no	none of the
1	0	0	0	hospitalise	medication	no	none of the
0	0	1	0	hospitalise	surgery and	no	
0	0	0	1	hospitalise	medication	no	none of the
1	0	0	0	hospitalise	medication	no	none of the
0	0	1	0	hospitalise	medication	no	
1	0	0	0	hospitalise	medication	no	none of the
0	0	1	0	hospitalise	medication	no	none of the
0	0	1	0	hospitalise	medication	no	none of the

0	0	0	1	hospitalise	medication	no		HIV/AIDS
1	0	0	0	hospitalise	medication	no		none of the
0	1	0	0	hospitalise	surgery and	no		
1	0	0	0	hospitalise	medication	no		none of the
1	0	0	0	hospitalise	medication	no		none of the
0	0	0	1	hospitalise	surgery and	no		none of the
1	0	0	0	hospitalise	medication	no		HIV/AIDS
1	0	0	0	hospitalise	medication	no		
1	0	0	0	hospitalise	surgery and	no		none of the
0	1	0	0	hospitalise	surgery and	no		none of the
1	0	0	0	hospitalise	medication	no		none of the
0	0	0	1	hospitalise	medication	no		none of the
0	1	0	0	hospitalise	medication	no		none of the
0	0	1	0	hospitalise	medication	no		none of the
				hospitalise	medication	no		none of the
1	0	0	0	hospitalise	medication	no		none of the
1	0	0	0	hospitalise	surgery and	no		hypertensio
1	0	0	0	hospitalise	surgery and	no		none of the
0	1	0	0	hospitalise	medication	no		none of the
0	1	0	0	hospitalise	medication	no		none of the
0	1	0	0	hospitalise	medication	no		none of the
0	0	0	1	hospitalise	medication	no		none of the
0	0	0	1	hospitalise	medication	no		none of the
0	1	0	0	hospitalise	medication	no		none of the
0	0	1	0	hospitalise	medication	yes	meningitis	none of the
0	1	1	0	hospitalise	surgery and	no		none of the
0	1	0	0	hospitalise	surgery and	no		HIV/AIDS
0	1	0	0	hospitalise	medication	no		none of the
0	1	1	0	hospitalise	surgery and	no		none of the
1	0	0	0	hospitalise	surgery and	no		none of the
0	0	0	1	hospitalise	medication	no		none of the
1	0	0	0	hospitalise	medication	no		none of the
1	0	0	0	hospitalise	medication	no		none of the
0	0	0	1	hospitalise	medication	no		hypertensio
0	0	1	0	hospitalise	medication	no		none of the

was it diagr	are you on	for what co	for how lor	what medic	form filled	participant	visuospatia	naming /3
above	yes				moca	26	4	3
above	yes				moca	27	4	3
above	yes				moca-B			
above	yes				moca-B			
above	no				moca-B			
above	no				moca-B			
above	no				moca	32	5	3
above	no				moca	32	5	3
above	no				moca	33	4	3
above	no				moca-B			
above	yes	asthma		salbutamol	moca-B			
above	no				moca	8	5	3
above	no				moca-B			
above	no				moca-B			
above	no				moca-B			
above	no				moca	11	5	3
above	no				moca-B			
above	no				moca	16	5	3
above	no				moca-B			
above	no				moca-B			
no	yes	HIV			moca-B			
above	no				moca	151	5	3
above	no				moca-B			
above	no				moca-B			
above	no				moca-B			
above	no				moca-B			
above	no				moca-B			
above	no				moca	144	4	3
no	no				moca-B			
above	no				moca-B			
above	no				moca-B			
yes	yes	HIV	12		moca-B			
no	no				moca-B			
yes	yes	HTN	2	dont know	moca-B			
above	no				moca-B			
above	no				moca-B			
above	no				moca-B			
above	no				moca	193	4	3
above	no				moca	190	5	3
above	no				moca-B			
above	no				moca-B			
above	no				moca-B			
yes	yes	HIV	6	DLT	moca-B			
above	no				moca-B			
above	no				moca-B			
above	no				moca-B			
above	no				moca-B			
above	no				moca-B			
above	no				moca	34	4	3
above	no				moca-B			
above	no				moca-B			

above	no				moca-B			
	no				moca-B			
no	yes	Hypertensi	2		moca-B			
above	no				moca-B			
yes	yes	HIV	16		moca-B			
above	no				moca-B			
above	no				moca-B			
above	no				moca-B			
above	no				moca-B			
above	no				moca-B			
above	no				moca-B			
above	no				moca-B			
no	no				moca-B			
no	no				moca-B			
above	no				moca-B			
no	yes	HIV		ARVs	moca-B			
above	no				moca-B			
no	no				moca-B			
no	yes	HIV	2	ARVS	moca-B			
above	no				moca-B			
above	no				moca	19	4	3
above	no				moca	18	5	3
above	no				moca-B			
above	no				moca-B			
above	no				moca-B			
above	no				moca-B			
above	no				moca-B			
above	no				moca-B			
above	no				moca	11	5	3
above	no				moca-B			
yes	yes	HIV	5	ARVS	moca-B			
above	no				moca	14	3	3
above	no				moca-B			
above	no				moca-B			
above	no				moca-B			
above	no				moca-B			
above	yes	KIDNEY STC	1	TRAMADOL	moca-B			
above	no				moca	39	3	3
above	no				moca	40	5	3
above	no				moca-B			
above	no				moca-B			
above	no				moca	43	3	3
no	yes	HIV	8	ARVS	moca-B			
above	no				moca-B			
above	no				moca-B			
above	no				moca-B			
above	no				moca	48	2	3
above	no				moca-B			
no	yes	HTN	1	nfelipine	moca-B			
above	no				moca	182	5	3
no	no				moca-B			

yes	yes	HIV	19	ARVS	moca-B			
above	no				moca	158	5	3
above	no				moca-B			
above	no				moca	160	5	3
above	no				moca-B			
above	no				moca-B			
above	no				moca-B			
above	no				moca-B			
above	no				moca-B			
above	no				moca-B			
yes	yes	HIV		ARV	moca-B			
yes	yes	HIV		ARV	moca-B			
above	no				moca-B			
yes	yes	HIV and hypertension		ARVs and ar	moca-B			
yes	yes	HIV		ARVs	moca-B			
above	no				moca-B			
above	no				moca	20	5	3
above	no				moca-B			
above	no				moca-B			
above	no				moca-B			
above	no				moca-B			
	yes	HIV		ARV	moca-B			
above	no				moca-B			
no	no				moca-B			
above	no				moca-B			
above	no				moca-B			
above	no				moca-B			
no	no				moca-B			
above	no				moca-B			
no	no				moca-B			
above	no				moca-B			
above	no				moca	101	4	3
above	no				moca-B			
above	no				moca-B			
above	no				moca-B			
above	no				moca-B			
above	no				moca-B			
yes	yes	Diabetes and HIV		ARVs and di	moca-B			
above	no				moca-B			
above	no				moca-B			
above	no				moca-B			
no	no				moca-B			
above	no				moca-B			
above	no				moca	110	3	3
above	no				moca-B			
no	no				moca-B			
above	no				moca-B			
above	no				moca-B			
no	no				moca-B			
above	no				moca-B			
above	no				moca-B			
above	no				moca-B			

yes	yes	HIV		ARVs	moca-B			
above	no				moca	608	4	3
no	no				moca-B			
above	no				moca-B			
above	no				moca-B			
above	no				moca-B			
no	yes	HIV	9	ARV	moca-B			
no	no				moca-B			
above	no				moca-B			
above	no				moca	600	1	3
above	no				moca-B			
above	no				moca-B			
above	no				moca-B			
above	no				moca-B			
above	no				moca-B			
above					moca-B			
yes	yes	Hypertensi	3		moca-B			
above	no				moca-B			
above	no				moca-B			
above	no				moca-B			
above	no				moca-B			
above	no				moca-B			
above	no				moca-B			
above	no				moca-B			
above	no				moca-B			
above	no				moca-B			
no	yes	HIV	27	ARVs	moca-B			
above	no				moca-B			
above	no				moca-B			
above	no				moca-B			
above	no				moca-B			
above	no				moca-B			
above	no				moca-B			
yes	no				moca-B			
above	no				moca-B			

list of digits	list of letter	serial 7 sub	language re	language flu	abstraction	delayed rec	orientation	total /30
1	1	3	2	1	2	5	6	27
0	1	3	1	0	2	5	6	28
0	1	1	2	0	2	2	5	20
1	1	3	1	1	2	2	6	25
1	1	0	0	0	2	1	6	18
1	1	3	1	0	2	2	6	24
1	1	3	2	1	2	4	6	28
2	1	1	1	0	1	3	6	23
2	1	2	2	0	2	4	6	27
0	1	2	1	1	2	4	6	24
2	1	3	1	1	2	4	6	27
1	1	3	1	1	2	0	6	23
2	1	3	0	0	1	4	6	24

2	1	3	0	0	2	4	6	25
1	1	1	1	0	2	4	5	23
2	1	2	2	0	2	5	6	28
1	1	3	1	0	2	4	6	24
1	1	1	0	0	2	2	6	19
1	1	2	2	0	1	5	6	26
0	0	0	2	0	2	3	6	19
1	1	3	0	0	2	1	6	20
1	1	3	2	1	2	2	6	26

0	1	3	2	0	2	3	4	23
2	1	3	0	1	2	5	6	28
1	1	3	2	1	2	2	6	26
2	1	3	1	1	2	3	6	26
0	1	1	1	0	2	3	6	20

1	1	3	2	0	2	1	6	23
0	1	0	0	0	2	1	3	11

Participant	b_executiv	b_fluency/	b_orientati	b_calculati	b_abstracti	b_delayedr	b_visuoper	b_naming /
28	0	0	6	3	2	3	3	4
29	0	2	6	1	3	4	2	4
30	0	1	6	3	2	3	1	4
31	0	0	6	3	2	3	1	4
157	1	0	6	3	3	3	3	4
13	0	1	6	2	3	2	3	4
15	1	2	6	3	3	5	3	4
7	0	0	6	3	3	4	2	4
9	1	0	6	1	3	2	1	4
12	0	0	6	1	3	2	2	4
25	1	0	6	3	0	5	3	4
24	0	1	6	3	2	5	1	4
149	0	1	6	3	3	4	3	4
152	0	0	6	3	3	3	2	4
153	0	0	6	1	2	0	0	4
120	0	0	6	3	1	5	3	4
118	1	1	6	3	3	5	2	4
180	1	0	6	3	3	5	2	4
105	0	0	5	1	2	2	0	4
111	1	1	6	3	3	1	1	4
108	1	1	6	3	3	1	1	4
100	0	0	6	3	2	4	2	4
102	0	0	6	3	1	5	0	4
23	1	1	6	3	3	2	3	4
22		0	6	1	3	5		
192		1	6	3	3	5	1	4
191		0	5	1	3	4	0	4
189	0	1	6	1	3	4	2	4
195		0	4	0	3	0		
188	0	1	6	2	3	3	2	4
187	0	1	6	2	3	4	2	4
186		0	6	0	1	2	0	2
185	0	0	4	2	2	3	1	4
184	0	1	6	3	3	5	1	4
36		2	6	3	1	3	0	4
35		2	6	3	3	4	2	4
148	1	1	6	3	3	5	3	4
140	1	0	6	1	3	3	3	4

139	0	0	6	0	1	1	0	4
2	1	0	6	3	3		1	4
116	1	1	5	0	3	0	0	4
3	0	0	5	2	2	3	0	3
113	1	1	6	3	3	5	3	5
122	0	1	5	0	0	0	1	4
1	1	1	6	3	3	3	2	4
125		0	4		3	0	0	4
137	1	0	6	2	3	5	2	4
136	0	0	6	4	3	5	3	4
126	1	1	6	3	3	5	3	4
4	0	0	2	2	1	0	0	3
6	1	0	6	3	3	2	2	4
128	0	1	6	2	3	4	1	4
130	0	0	6	2	3	3	1	3
132	1	0	6	2	3	1	1	4
5	1	0	6	3	3	3	1	4
14	1	0	6	3	3	4	2	3
21	0	0	5	1	1	0	2	4
21	0	1	3	3	3	5	3	4
17	1	1	6	3	3	5	2	4
16	1	0	6	2	2	4	3	4
7	0	0	6	2	3	4	1	4
8	0	0	5	3	3	1	0	4
9		0	4	1	3	3	2	4
10	1	1	6	3	3	4	3	4
0	0	6	6	3	1	3	2	4
13	0	0	6	3	3	5	3	4
15	0	0	6	1	3	2	1	4
155		0	5	2	3	4	2	4
154		0	4	1	1	3	0	4
156	1	0	6	3	3	4	2	4
0	0	6	3	2	1	1	2	4
41		0	6		3	5	1	4
42	1	1	6	3	3	5	2	4
44	1	2	6	3	3	5	2	4
45	0	1	6	3	3	2	2	4
46	0	1	6	3	3	2	2	4
47	0	1	6	2	3	4	3	4
146	1	0	6	3	3	5	3	4
181	0	0	5	1	1	3	2	4
196	0	1	6	1	3	3	1	4

194	0	1	6	3	3	2	2	3
37		2	5	3	3	4	2	4
104	1	1	6	3	3	4	0	4
162	0	1	6	3	3	3	0	4
161	1	0	6	3	3	0	1	4
163	0	2	6	3	3	4	2	4
164	0	0	6	3	3	1	0	2
165	1	0	6	3	3	3	2	4
166	1	1	6	3	3	3	2	4
168	1	1	6	3	3	3	3	4
169	1	1	6	3	3	4	3	4
167	1	1	6	3		5	2	4
27	1	0	6	3	3	3	2	4
17	1	0	6	3	3	2	2	4
19	0	1	6	3	3	2	2	4
18	0	0	6	2	3	1	2	4
21	1	1	6	3	3	5	3	4
22	1	1	6	3	3	5	2	4
23	1	1	6	3	3	5	3	4
24	1	2	6	3	3	3	2	4
28	0	1	6	1	3	4	2	4
141	0	0	6	3	3	5	1	4
28	1	1	6	3	3	5	3	4
26	1	1	6	3	3	5	2	5
30	1	0	6	3	3	5	2	4
31	1	1	6	3	3	3	3	4
29	1	0	6	3	3	3	2	4
107	1	0	6	3	3	4	2	4
121	1	1	6	2	3	3	0	4
124	1	0	6	0	3	5	0	4
123	0	1	6	1	3	4	0	4
127	1	1	6	3	3	4	1	4
129	0	1	6	0	3	4	3	4
133	0	0	6	3	3	3	2	4
143	1	0	6	3	3	4	3	4
142	0	1	6	3	3	4	0	4
145	1	1	6	3	3	3	2	4
147	1	0	6	3	3	4	1	4
112	1	1	6	3	3	5	2	4
103	0	1	6	2	3	5	0	4
114	1	1	6	0	3	4	4	4
115	1	1	6	3	2	4	0	4
117	1	2	6	3	3	5	2	4
119	1	1	6	3	3	4	2	4
131	0	1	6	3	3	4	2	4
138	0	0	6	3	3	0	0	4
135	0	1	6	3	3	4	1	4

134	1	0	6	3	3	4	2	4
607	0	0	6	1	3	5	1	3
606	0	1	6	1	3	5	2	4
605	0	1	5	0	3	3	1	4
604	0	0	5	0	2	10	1	4
604	0	0	2	0	3	1	0	3
602	0	0	3	3	3	4	1	4
601	0	1	6	0	3	4	0	4
609	0	0	4	3	3	4	1	2
714	0	0	6	1	3	4	0	4
714	0	1	6	0	3	0	0	4
712	0	1	6	1	3	4	0	3
613	0	0	6	0	3	5	2	4
612	0	0	4	2	3	4	1	3
611	0	0	5	3	3	5	2	4
610	0	0	5	0	3	2	2	4
712	0	0	3	0	3	2	0	3
711	0	1	6	1	2	3	2	4
710	0	0	6	2	2	4	2	4
709	1	1	6	1	3	5	3	4
708	0	0	6	1	3	4	0	4
707	0	1	6	2	3	5	2	4
706	0	1	6	0	3	5	3	4
705	0	1	6	1	3	5	3	4
704	0	0	5	1	3	4	0	4
703	0	0	6	1	3	1	0	4
702	0	0	6	2	3	4	2	4
701	0	1	6	1	3	4	2	4
700	0	0	3	0	3	4	1	4
25	1	0	6	3	0	5	3	4
24	0	1	6	3	2	5	1	4
23	1	1	6	3	3	2	3	4
22	0	0	6	1	3	5	0	0

number in	number in	b_total/30
1	2	24
0	0	22
1	2	23
1	2	22
1	1	26
1	2	24
1	2	30
1	2	25
1	2	21
0	0	18
1	2	25
1	1	24
1	2	27
1	2	26
0	0	13
1	2	26
1	2	28
1	2	27
0	0	13
1	2	23
1	2	23
1	2	24
0	0	19
1	2	26
		19
		19
1	2	25
		7
0	2	24
1	2	26
		10
1	0	19
1	2	26
		17
		24
1	1	29
1	2	25

1	1	16
0	0	18
		13
1	0	16
1	3	29
1	2	14
1	2	26
		11
1	2	26
1	1	27
1	2	28
0	0	8
0	2	26
1	2	24
1	1	20
1	2	21
1	2	24
1	1	25
1	2	28
1	2	28
1	2	25
0	2	22
1	2	19
1	2	28
1	2	22
1	2	27
1	2	18
1	2	22
1	2	26
1	2	21
1	2	28
1	2	29
1	2	24
1	2	24
1	2	26
1	2	28
1	2	19
1	2	21

1	2	23
1	2	25
1	2	24
1	2	23
1	2	27
1	2	18
1	2	25
1	2	26
2	2	27
1	2	28
1	2	28
1	2	25
1	2	24
1	2	24
1	2	21
1	2	29
1	2	28
1	2	29
1	2	25
1	2	24
1	1	24
1	2	29
1	3	28
1	3	27
1	2	27
1	2	29
1	2	26
1	2	23
1	2	21
1	2	23
1	2	26
1	2	24
1	3	24
1	2	26
1	2	24
1	2	26
1	3	25
1	2	28
1	2	24
1	2	25
1	2	24
1	1	30
1	2	27
1	2	26
1	2	19
1	2	25

1	2	26
0	0	19
1	2	25
0	1	18
0	0	12
0	0	9
0	2	20
1	2	21
0	0	17
1	2	21
1	2	17
0	0	18
1	2	23
0	0	17
1	2	26
1	2	19
0	0	12
1	2	22
1	2	23
1	2	27
0	0	19
1	2	26
1	2	25
1	2	26
0	0	18
0	1	17
1	2	24
1	2	24
0	0	16
1	2	25
1	1	24
1	2	26
0	0	15
